# Health service costs and clinical gains of psychotherapy for personality disorders: a randomized controlled trial of day-hospital-based step-down treatment versus outpatient treatment at a specialist practice

**DOI:** 10.1186/1471-244X-13-315

**Published:** 2013-11-22

**Authors:** Elfrida Hartveit Kvarstein, Espen Arnevik, Vidar Halsteinli, Frida Gullestad Rø, Sigmund Karterud, Theresa Wilberg

**Affiliations:** 1 Department of Personality Psychiatry, Oslo University Hospital, Kirkeveien 166, 0407 Oslo, Norway; 2 Department of Psychology, University of Oslo, P.b. 1094 Blindern, 0317 Oslo, Norway; 3 Norwegian University of Science and Technology, NTNU, Faculty of Medicine, 7489 Trondheim, Norway; 4 Institute of Clinical Medicine, University of Oslo, Pb 171 Blindern, 0318 Oslo, Norway

**Keywords:** Psychotherapy, Personality disorders, Health service costs

## Abstract

**Background:**

Day-hospital-based treatment programmes have been recommended for poorly functioning patients with personality disorders (PD). However, more research is needed to confirm the cost-effectiveness of such extensive programmes over other, presumably simpler, treatment formats.

**Methods:**

This study compared health service costs and psychosocial functioning for PD patients randomly allocated to either a day-hospital-based treatment programme combining individual and group psychotherapy in a step-down format, or outpatient individual psychotherapy at a specialist practice. It included 107 PD patients, 46% of whom had borderline PD, and 40% of whom had avoidant PD. Costs included the two treatment conditions and additional primary and secondary in- and outpatient services. Psychosocial functioning was assessed using measures of global (observer-rated GAF) and occupational (self-report) functioning. Repeated assessments over three years were analysed using mixed models.

**Results:**

The costs of step-down treatment were higher than those of outpatient treatment, but these high costs were compensated by considerably lower costs of other health services. However, costs and clinical gains depended on the type of PD. For borderline PD patients, cost-effectiveness did not differ by treatment condition. Health service costs declined during the trial, and functioning improved to mild impairment levels (GAF > 60). For avoidant PD patients, considerable adjuvant health services expanded the outpatient format. Clinical improvements were nevertheless superior to the step-down condition.

**Conclusion:**

Our results indicate that decisions on treatment format should differentiate between PD types. For borderline PD patients, the costs and gains of step-down and outpatient treatment conditions did not differ. For avoidant PD patients, the outpatient format was a better alternative, leaning, however, on costly additional health services in the early phase of treatment.

**Trial registration:**

Clinical Trials NCT00378248

## Background

Personality disorders (PD) have been associated with considerable social and occupational impairment, high medical comorbidity, extensive health service use, and low quality of life [1-9]. Relative to other medical and psychiatric conditions, the personal and economic burden of PD is severe [10,11]. The choice of an optimal treatment format for poorly functioning PD patients has therefore high clinical and societal relevance.

Intensive hospital-based treatment programmes, most often day-hospital and step-down formats, are costly. However, several studies have indicated that these treatment formats yield both clinical improvements and effective prevention of suicidal crises [12-17]. The high costs of specialized, intensive treatments may then be outweighed by reduced emergency costs, and the programmes can be recommended as cost-effective treatments of PD.

The day-hospital-based step-down programmes represent well-established, long-term treatment alternatives for PD patients and are frequently found feasible [18]. Group psychotherapy is a central component of these programmes and saves therapist resources. As patients with different PDs are often included in the same, standard programme, the treatments are also implementable in less populated, more rural areas.

Individual psychotherapy, provided by specialists in outpatient practice, may be a relevant treatment alternative, presumably less intensive, and if so, also cheaper. Clinical benefits of outpatient psychotherapy have been demonstrated [19-21], but few have investigated the associated economic aspects and health service use. Low-intensity outpatient treatments can be an option for patients with milder personality pathology, but are usually considered insufficient for poorly functioning PD patients [17,22].

Psychotherapy research has frequently focused on treatments designed specifically for borderline PD, and the cost-effectiveness of several approaches has been investigated [23,24]. As yet, studies of specific treatments for avoidant PD are scarce. There is, however, a growing concern about the severity of this disorder [25-27], and in two large cost-effectiveness studies, naturalistic patient samples in outpatient, day-hospital, or inpatient treatments have been investigated. The first study included patients with cluster B PDs [28], and the second, cluster C PDs [29]. The results of these studies suggest that the cost-effectiveness of different treatment formats differs considerably for patients with different personality pathology, favouring intensive formats for PDs within cluster C.

The present study aims, firstly, to compare costs and clinical gains for PD patients randomly allocated to two different formats of psychotherapy: (1) An intensive, day-hospital-based treatment in a step-down format and (2) individual psychotherapy in specialist outpatient practice, and secondly, to specifically investigate the differences associated with two frequent PD subgroups, borderline and avoidant PD. The study is a part of the Ullevål Personality Project (UPP).

## Methods

### The day-hospital-based step-down treatment

The step-down condition (SDC) had a maximum treatment duration of four years with phases of different intensity: (1) Day-hospital programme with psychodynamic and cognitive behavioural group therapies (18 weeks), followed by (2) outpatient therapy, with weekly conjoint individual and group psychotherapy during the first 2.5 years and (3) a final year of outpatient group psychotherapy alone. The psychotherapy groups were conducted by experienced day-hospital staff, 75% of whom had five-year group analytic training. The individual therapists were experienced, practising psychotherapists.

### The treatment in specialist outpatient practice

The treatment given in outpatient practice (OPC) had no limitations of therapy duration, intensity, or use of other health services. It was given in accordance with each therapist’s preferred method and practice. The OPC therapists were experienced, practising psychologists or psychiatrists, and the majority reported adherence to psychodynamic or psychoanalytic treatment approaches.

### Participants

The study recruited naturally treatment-seeking patients who had been referred to day-hospital treatment. Exclusion criteria were based on conditions not normally treated in day-hospital treatment: schizotypal PD, antisocial PD, substance dependency, psychosis, organic syndromes, bipolar I, untreated attention-deficit hyperactivity, or pervasive developmental disorder. Patients were assessed before random assignment to SDC or OPC. An independent randomization co-ordinator was responsible for randomization procedures (computer-generated random numbers, Statistical Package for the Social Sciences, SPSS) [30]. Additional file 1: Chart 1 demonstrates the numbers of patients who were excluded, who withdrew, or who missed assessments. Statistical analyses included a total of 107 patients (*n*_SDC_ = 56 and *n*_OPC_ =51). Mean age of this sample was 31 years (SD = 7), and 76% of the patients were female.

### Baseline diagnostic assessments

Standardised, semi-structured diagnostic interviews for Axis-I and -II disorders were conducted before randomisation: (1) the Mini-International Neuropsychiatric Interview (M.I.N.I.) version 4.4 for Axis-I diagnoses [31]; and (2) the Structured Clinical Interview for DSM Disorders (SCID-II) for Axis-II diagnoses [32]. Clinical day-hospital staff trained for diagnostic procedures performed the interviews. The reliability of Axis-II diagnoses was confirmed by an independent observer who rated 24 videotaped SCID-II interviews. The kappa values for avoidant and borderline PD were 0.75 and 0.66, respectively. Table 1 shows baseline Axis-I and -II diagnoses for the 107 patients included in longitudinal analyses.

**Table 1:** Axis-I and -II disorders at baseline

	**SDC (*n*** **= 56)**	**OPC (*n*** **= 51)**
*Axis-II disorders*	*Number (%)*	*Number (%)*
*Most frequent*		
Borderline	27 (47)	24 (46)
Avoidant	26 (45)	18 (35)
*Comorbidity*		
Borderline and Avoidant	10 (17)	6 (12)
Borderline, not Avoidant	17 (33)	18 (35)
Avoidant, not Borderline	16 (31)	12 (22)
*Other Axis-II disorders*		
Schizoid	1 (2)	0
Paranoid	10 (17)	6 (12)
Narcissistic	0	2 (4)
Obsessive-compulsive	7 (12)	3 (6)
Dependent	4 (7)	3 (6)
Axis-II not otherwise specified	10 (17)	13 (25)
*Axis-I disorders*		
Mood	49 (85)	46 (89)
Anxiety	52 (90)	44 (85)
Obsessive-compulsive	9 (16)	4 (8)
Somatoform	6 (10)	5 (10)
Eating	6 (10)	9 (17)
Substance abuse/dependency	11 (19)	17 (33)
Post-traumatic stress disorder	5 (9)	5 (10)
*Severity of the disorder*	Mean (SD)	Mean (SD)
Number of Axis-I disorders	3.5 (1.4)	3.3 (1.5)
Number of Axis-II disorders	1.5 (0.7)	1.3 (0.6)
Number of Axis-II criteria	14.9 (6.3)	14.8 (5)
Quality of life	3.3 (1.6)	3.6 (1.7)
Symptom distress (SCL-90-R, global index)	1.7 (0.7)	1.8 (0.5)
Interpersonal problems (CIP)	1.7 (0.5)	1.7 (0.5)

Standard deviation (SD). Step-down condition: SDC, Outpatient condition: OPC.

### Assessments of health-service use

Information on health-service use and medication was collected through UPP-designed interviews and questionnaires. Reports of health-service use included (1) psychotherapeutic treatment (outpatient clinic, practising specialist, and psychotherapeutic day hospital), (2) emergency health services (psychiatric and general outpatient emergency services, admissions to psychiatric and medical hospitals, emergency outpatient and inpatient treatment at mental health centres and addiction clinics), (3) general practitioner (GP) visits, (4) community services (psychiatric community nurse, community day centre, and social welfare co-ordinator visits), (5) other specialist services (outpatient, day-patient or inpatient treatment at an addiction clinic or day-patient treatment at a psychiatric hospital), and (6) pharmacological treatment. The sum of health services included those listed in points 1–6.

Use of health services and medication was reported retrospectively for four time periods: The year preceding randomisation (period 0), the initial 0–8 months of the trial (period 1), the middle 9–18 months (period 2), and the final 19–36 months (period 3). Table 2 demonstrates the frequencies of different health services used.

**Table 2:** Health-service use and occupational functioning

	***The year before the trial***			***First 0–8 months of trial***						***Last 19–36 months of trial***					
	***% full sample***			***% OPC***			***% SDC***			***% OPC***			***% SDC***		
	***All PDs***	***APD***	***BPD***	***All PDs***	***APD***	***BPD***	***All PDs***	***APD***	***BPD***	***All PDs***	***APD***	***BPD***	***All PDs***	***APD***	***BPD***
*Psychotherapy*															
Outpatient clinic	58	56	76	44	50	47	56	50	59	20	0	27	82	92	79
Practising specialist	25	28	16	80	90	71	56	64	47	54	75	40	36	50	36
Psychotherapeutic day hospital	6	8	8	17	30	12	98	100	94	0	0	0	0	0	0
*Emergency services*															
Emergency outpatient services	41	28	56	28	10	24	21	21	18	37	25	40	34	8	50
Psychiatric inpatient services	24	24	36	17	20	12	12	14	12	6	0	7	16	17	7
Medical inpatient services	17	8	28	11	0	18	12	7	18	23	25	20	11	8	14
*GP and community services*															
General practitioner	90	84	92	79	75	78	78	63	94	97	100	93	91	75	93
Community healthcare/social services	35	20	56	54	70	47	42	43	35	54	63	53	71	67	79
Social welfare coordinator	28	14	44	49	57	35	39	43	29	53	70	53	55	35	71
*Other specialist services*															
Addiction clinic, outpatient	9	8	12	13	20	12	0	0	0	0	0	0	5	8	7
Psychiatric hospital, day patient	14	12	20	2	7	0	0	0	0	0	0	0	2	0	7
*Pharmacological treatment*	61	56	68	64	70	59	64	71	71	33	37	33	50	42	54
	***Start of the trial***			***8-month assessment***						***36-month assessment***					
	***% full sample***			***% OPC***			***% SDC***			***% OPC***			***% SDC***		
	***All PDs***	**APD**	**BPD**	***All PDs***	**APD**	**BPD**	***All PDs***	**APD**	**BPD**	***All PDs***	**APD**	**BPD**	***All PDs***	**APD**	**BPD**
*Occupational functioning*															
Present employment/education	49	40	59	58	50	81	49	50	50	80	87	70	61	58	70

Health-service use and occupational functioning (%) is presented for the full sample (all PDs), and for subgroups of PD patients with avoidant (APD), and not comorbid borderline (BPD), and BPD, not APD during period 0 (the year before randomisation), period 1 (the first eight months of the trial), and period 3 (the last 19–36 months of the trial).

### Estimation of health service costs

Calculations of mean monthly costs were based on the individual patients’ reported health service use in each assessment period and the standard costs of each specific service. Standard costs of day-hospital treatment were estimated using annual accounts from the Department of Personality Psychiatry, Oslo University Hospital. Costs per outpatient consultation in OPC and SDC were based on reports of annual activity and income for the participating therapists. Costs related to treatment at mental health centres, medical and psychiatric hospitals, addiction clinics, outpatient general and psychiatric emergency services, and GPs were obtained from published reports [33-36]. Costs related to community and social services were based on information from one large municipality. Medication costs were based on information from the Norwegian Medicines Agency. All unit costs are from the year 2006 and are presented in euros (Table 3). The exchange rate was 1 euro = 8 Norwegian krone (NOK). Table 4 demonstrates the mean monthly sum of health service costs per patient for each assessment period.

**Table 3:** Standard costs of health services

	***Cost per consultation/day €***
*Psychotherapy*	
Outpatient clinic	118
Practising specialist	110
Psychotherapeutic day hospital	165
*Emergency services*	
Emergency outpatient services	101
Psychiatric inpatient services	644
Medical inpatient services	1077
Psychiatric hospital, day patient	445
*GP*	
General practitioner	33
*Community services*	
Community healthcare/social services	48
Social welfare coordinator	28
Mental health centre, day patient	309
*Addiction treatment*	
Addiction clinic, outpatient	118
Addiction clinic, day patient	309

**Table 4:** Mean costs and psychosocial functioning

	**Step-down condition**				**Outpatient condition**			
	**Costs**			**GAF**	**Costs**			**GAF**
	***n***	**Mean (SD)**	**Median**	**Mean (SD)**	***n***	**Mean (SD)**	**Median**	**Mean (SD)**
Costs: The year before trial	45	844 (1793)	160	47 (4)	46	1406 (2604)	370	48 (5)
GAF: 0 months								
Costs: First 0–8-month period	52	1462 (854)	1325	51 (10)	46	1294 (3034)	536	50 (12)
GAF: 8 months								
Costs: 9–18-month period	47	618 (806)	496	53 (10)	37	944 (1771)	368	57 (12)
GAF: 18 months								
Costs: Last 19–36-month period	45	794 (1128)	474	57 (12)*	36	407 (620)	270	67 (13)*
GAF: 36 months								

The table demonstrates mean monthly costs (€) per patient (treatment and additional health services) for each assessment period and global functioning (GAF) scores at baseline (0) and after 8, 18, and 36 months. Statistically significant differences between the outpatient and step-down condition are marked with an asterisk (* = *P* < 0.05, independent-samples *t-*tests).

### Assessments of functioning

Global functioning (GAF; Axis V, DSM-IV, American Psychiatric Association) was evaluated at the baseline assessment and at all follow-up assessments (after 8, 18, and 36 months). The observer-rated GAF provides a composite score of psychosocial functioning on a 0–100 scale. Higher GAF scores indicate better psychosocial functioning, and a level of 60 represents the cut-off point between satisfactory functioning/mild impairment and moderate/severe impairment. Baseline GAF was rated by day-hospital staff [37], and ratings at 8-, 18-, and 36-month time points were performed by research fellows. GAF reliability was tested using independent raters. The reliabilities (ICC (2,1): intra-class correlation coefficients, two-way, single, random measures) for GAF scores were 0.56 at baseline (confidence interval, CI: 0.02–0.83), 0.81 at 8 months (CI: 0.51–0.92), 0.85 at 18 months (CI: 0.59–0.94), and 0.94 at 36 months (CI: 0.88–0.97). Table 4 shows mean GAF scores at all assessments. Occupational status was assessed using a questionnaire designed for UPP.

### Completeness of data

The average number of assessments per patient over the study period was 3.3 (SD = 0.9), with equal frequencies in SDC and OPC. Fifty-four percent of the patients (*n* = 59) responded at all four assessments (OPC = 57%, SDC = 50%), while 26% (*n* = 29) were assessed three times (OPC = 15%, SDC = 36%), and 15% (*n* = 16) were assessed twice (OPC = 24%, SDC = 7%). One patient in OPC and two in SDC had only one assessment.

### Informed consent

The UPP was approved by the Norwegian Data Inspectorate and the Regional Committee for Medical Research Ethics. Participation was obtained with written informed consent.

### Statistical procedures

Longitudinal data were statistically analysed by mixed models (SPSS, version 19). Model fit was decided by comparing log likelihood statistics (Aikikes Information Criterion, AIC).

A piecewise change model [38] combining periods 0–1 (linear curves) and periods 1–3 (linear or quadratic curves) with a knot at period 1 gave the best approximation to the data. For periods 1–3, quadratic curves were chosen to model the longitudinal change of the health service cost variables: treatment, emergency services, and the sum of all health service costs, while linear curves were chosen for the other cost variables and GAF scores. In addition, a linear change model included only periods 0 and 3. Unstructured covariance was chosen for the repeated measures. Such covariance minimizes the risk for type I errors [39] and gave the best model fit.

The main predictor analyses investigated the longitudinal effect of treatment condition on health service costs and global functioning. The effects of the diagnostic variables were investigated as predictors and as moderators. The main moderator analyses included the baseline interactions: treatment condition × PD (thus controlling for baseline variation) and the three-way interaction with time: treatment condition × PD × time. In addition, all analyses included models testing the possible effects of comorbidity with other Axis-I or -II disorders.

In this study, 16 patients had both borderline and avoidant PD, leaving 28 and 35 patients with avoidant or borderline PD, respectively. However, all statistical inferences in the moderator analyses are based on models including all patients (*n* = 107), models with patients who had at least three assessments (*n* = 88), and models controlling for the separate effects of comorbidity.

Table 5 shows estimates of differences in the longitudinal course of costs: (A) treatment, (B) emergency services, and (C) the sum of health service costs for patients in the two treatment conditions and subgroups with borderline and avoidant PD. Table 6, section A, demonstrates differences in the longitudinal course of GAF. Mixed-model analyses of the other variables are described in the results section with the corresponding *P*-values denoted by *P*_*PWM*_ (piecewise model) and *P*_*LM*_ (linear model for period 0–3). Independent-samples *t-*tests were used for other comparisons, and when referring to these analyses, *P*-values are denoted by *P*_*TT*_.

**Table 5:** Estimated differences in costs for patients in step-down and outpatient treatment

		***∆ costs: first trial period (0*–*8months)***		***∆ change in costs per month during trial***				***∆ costs: entire trial period (36 months)***			***Model fit***
		***∆ mean estimate (SE)***	***P***	***∆ initial slope (SE)***	***P***	***∆ curvature (SE)***	***P***	***∆ grand mean (SE)***		***P***	***AIC***
*Costs per patient*											
*(A) Monthly costs of treatment*	∆ (SDC-OPC)	720 (69)	.00	−86 (7)	.00	2 (0.2)	.00	281 (33)		.00	4814
	*Predictor effects within OPC*										
	∆ (APD - other PDs)	264 (101)	.01	−19 (12)	ns	0.4 (0.3)	ns	136 (43)		.00	4813
	∆ (BPD - other PDs)	−11 (101)	ns	−8 (11)	ns	0.2 (0.3)	ns	61 (41)		ns	4819
*(B) Monthly costs of emergency services*	∆ (SDC-OPC)	−797 (500)	ns	45 (64)	ns	−0.4 (1.9)	ns	−331 (28)		.00	5541
	*Moderator effects*										
	∆ (SDC_APD_ - OPC_APD_)	−2411 (829)	.005	179 (109)	ns	−3.4 (3.3)	ns	−956 (120)		.00	5547
	∆ (SDC_BPD -_ OPC_BPD_)	−1451 (749)	ns	199 (95)	.04	−4.9 (2.8)	ns	−303 (86)		.00	5546
*(C) Sum of all monthly costs*	∆ (SDC-OPC)	10 (504)	ns	−53 (65)	ns	2 (2)	ns	7 (44)		ns	5625
	*Moderator effects*										
	∆ (SDC_APD_ - OPC_APD_)	−1717 (829)	.04	92 (109)	ns	−1.3 (3.5)	ns	−905 (147)		.00	5626
	∆ (SDC_BPD -_ OPC_BPD_)	−639 (755)	ns	94 (96)	ns	−2.1 (3.0)	ns	−37 (88)		ns	5632
								***Mean estimate (SE)***			***Incremental costs (SE)***
								***SDC***	***OPC***		
*(D) Sum of all costs over 36 months*	All patients							31823 (479)	31607 (956)	ns	
	*Moderator effects*										
	Predicted change: APD (not BPD)							35524 (381)	49728 (525)	.00	−14204 (633)
	Predicted change: BPD (not APD)							29709 (1275)	21309 (2482)	ns	

Table 5 shows mixed-model estimates of (A) treatment, (B) emergency, and (C) total (all health service) costs (€) in models with treatment included as a predictor (SDC: the step-down condition, OPC: the outpatient condition). Monthly costs in the first trial period, change per month for the 0–36 month trial period, and grand means for the whole trial period are presented as: (1) *∆ (SDC-OPC):* Differences (∆) between SDC and OPC. (2) *Predictor effects within OPC*: Cost differences for subgroups within OPC. (3) *Moderator effects*: Cost differences between SDC and OPC for subgroups of patients with (a) avoidant PD, APD: ∆ (SDC_APD_ - OPC_APD_) and (b) borderline PD, BPD: ∆ (SDC_BPD_ - OPC_BPD_). Estimates are presented as means with standard errors (SE). Akaikes Information Criterion (AIC) indicates goodness of fit for each model. Table 5 also shows (D): *The sum of all costs over 36 months:* Estimations of total 36-month costs and incremental costs (if significant SDC-OPC difference) based on mixed-model predicted values for all patients and subgroups with (a) avoidant and (b) borderline PD. *P*-values are presented if *p* < 0.05, ns indicates *p* > 0.05.

**Table 6:** Estimated differences in clinical gains for patients in step-down and outpatient treatment

		***∆ GAF at 0 months***		***∆ GAF-change rate per month during trial***		***Model fit***
		***∆ mean estimate (SE)***	***P***	***∆ Slope (SE)***	***P***	***AIC***
*(A) Global functioning (GAF)*	∆ (SDC-OPC)	1.3 (2.1)	ns	−0.4 (0.1)	.00	2586
	*Moderator effects*					
	∆ (SDC_APD_ - OPC_APD_)	1.6 (3)	ns	−0.5 (0.2)	.00	2583
	∆ (SDC_BPD -_ OPC_BPD_)	0.6 (3)	ns	−0.3 (0.2)	ns	2594
*(B) 36-month GAF change*		*∆ GAF (0-36-months) mean estimates (SE)*				*Incremental effects (SE)*
		*SDC*	*OPC*			
	All patients	10 (0.3)	18 (0.1)		.00	−8 (0.3)
	*Moderator effects*					
	Predicted change: APD (not BPD)	6 (0.5)	19 (0.01)		.00	−13 (1)
	Predicted change: BPD (not APD)	14 (0.6)	17 (0.2)		ns	

Table 6 shows mixed-model estimates of the Global Assessment of Functioning (GAF) in models with treatment included as a predictor (SDC: the step-down condition, OPC: the outpatient condition). (A): Mean estimated GAF (SE: standard error) at baseline (intercept) and change per month (linear slope) during the trial presented as (1) *∆ (SDC-OPC):* Difference (∆) between SDC and OPC, and (2) *Moderator effects*: (SDC-OPC) difference for subgroups of patients with (a) avoidant PD, APD: ∆ (SDC_APD_ - OPC_APD_) and (b) borderline PD, BPD: ∆ (SDC_BPD_ - OPC_BPD_). Akaikes Information Criterion (AIC) indicates goodness of fit for each model. (B): *36-month GAF change:* Estimations of 36-month GAF change and incremental effects (when a significant difference existed between SDC and OPC) based on mixed-model predicted values. *P*-values are presented if *p* < 0.05, ns indicates *p* > 0.05.

### Possible bias of missing data

It is recommended to investigate the possible bias from missing data by comparing subsamples of patients with different attrition patterns using mixed-model statistics [40]. The piecewise change model was therefore used to compare trajectories for: (1) patients with and without the last assessment (*n* = 81) and (2) patients with all four, three, and two assessments. Longitudinal change for the investigated subgroups did not differ significantly from the rest of the sample (all differences *P* > 0.05). Moreover, comparison of log likelihood statistics and residual variation indicated that the subsamples did not represent a notable source of systematic longitudinal variation. We therefore concluded that the bias was small.

### Extreme costs

The results of cost analyses were cross-checked in samples, excluding six patients (*n* = 101) with extreme cost values. These outliers included one OPC patient (avoidant PD) with high emergency costs because of extensive psychiatric hospitalisation and five patients with more than seven days of medical hospitalisation (four OPC patients: two with avoidant PD, two with borderline PD, and one SDC patient with borderline PD).

### Incremental costs and effects

The incremental cost per patient was defined as the difference in the sum of all health service costs over the whole 36-month trial period for SDC patients versus OPC patients (Table 5, section D). The incremental effect was defined as the difference in GAF improvement for SDC versus that of OPC over the same period (Table 6, section B). Calculations of both were based on mixed-models predicted values. The incremental cost per incremental effect indicates the extra cost per GAF point gained.

## Results

### Costs of the two specialist treatments

**Figure 1:**
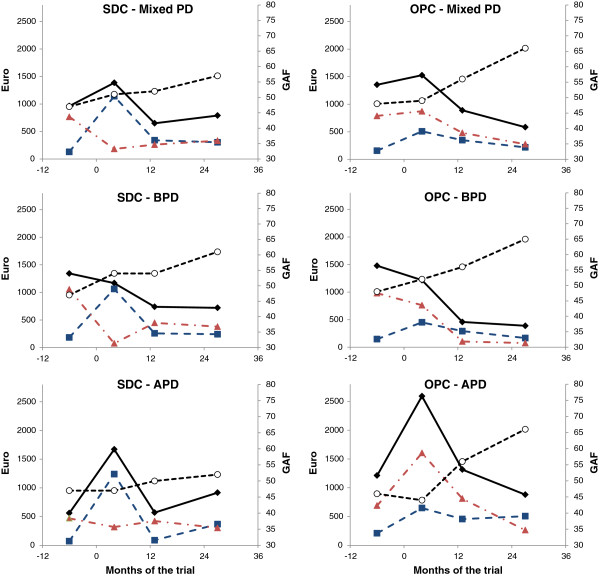
**Longitudinal change of health service costs and global functioning.** The figures illustrate the longitudinal course (mixed-model estimations) of health service costs (mean total health service costs: solid black line, mean treatment costs: blue dotted line, and mean emergency costs: red dotted line) with costs (in euros) given on the left Y-axis. The change trajectory for global functioning (given on the right Y-axis) is represented by the black dotted line. Separate figures demonstrate (1) the full sample of patients with different personality disorders (mixed PD) in the step-down (SDC) and the outpatient condition (OPC), (2) patients with borderline PD (BPD), and (3) with avoidant PD (APD) in the two treatment conditions. The point of randomisation is denoted by Time = 0 months.

Hospital-based, step-down treatment (SDC) was, in itself, more expensive than psychotherapy in specialist outpatient practice (OPC) (Table 5 section A). Initial day-hospital treatment in SDC (period 1) was the most costly (Figure 1). Although the shift from day-hospital to outpatient conjoint therapy (period 1 to period 2) represented a sharp cost decline, average monthly costs nevertheless remained higher than OPC throughout the trial.

In SDC, there was generally little variation in treatment costs and no significant deviation related to any PD subgroups (*P*_*PWM*_ > 0.1). In OPC, treatment costs varied more, and depended on the patient’s type of PD. For patients with avoidant PD, treatment costs were especially high in period 1 (Table 5, section A). Compared with other OPC patients, these patients reported more frequent individual therapy sessions [mean_APD_ 21 (SD 13) versus mean_other PDs_ 15 (SD 15), *P*_*TT*_ < 0.05] and a greater number of additional consultations at outpatient clinics, other day-hospital treatment, and addiction treatment (all *P*_*TT*_ < 0.05). Treatment costs in SDC and OPC were not influenced by comorbid mood or anxiety disorders, other specific PDs, or increasing numbers of Axis-I or -II disorders (*P*_*PWM*_ > 0.1).

### Additional health service costs during the trial

#### Emergency health service costs during the trial

Average monthly emergency costs over the trial period were generally higher in OPC than in SDC (Table 5, section B, grand means, and Figure 1). There was an overall emergency cost decline from baseline to 36 months in both treatment conditions (period 0–3: no significant difference, *P*_*LM*_ > 0.1), and a significant trend of more rapid initial cost reductions for borderline PD patients in SDC (Table 5, section B).

OPC patients with avoidant PD were associated with particularly high emergency service costs in period 1 (Table 5, section B and Figure 1) with more frequent use of inpatient psychiatric services (Table 2) and a higher number of inpatient days [mean number of days: 1 (SD 6) and 11 (SD 27), for SDC and OPC, respectively (*P*_*TT*_ < 0.05)]. The trend persisted in the model that excluded extreme outliers [mean number of days, OPC: 5 (SD 17)].

Increasing numbers of PDs were also associated with higher emergency costs in OPC [mean cost increase (euros) per PD: 910 (SE 328), *P*_*PWM*_ = 0.007]. In SDC neither PD subgroups nor increasing numbers of PDs were associated with deviating emergency costs during the trial (*P*_*PWM*_ >0.1). In both SDC and OPC, emergency costs were not significantly influenced by differences in Axis-I comorbidity (numbers of Axis-I disorders, or specific mood or anxiety disorders, *P*_*PWM*_ > 0.1).

#### Medication, GP, and community service costs during the trial

The average monthly costs of medication were higher in OPC than in SDC in period 1 (*P*_*PWM*_ < 0.05), but longitudinal change across periods 1–3 did not differ between treatment conditions (*P*_*PWM*_ > 0.1). Costs of GP consultations declined over time in both SDC and OPC (*P*_*PWM*_ > 0.01). Costs of community-based services were initially low, increased over time (*P*_*PWM*_ < 0.05), and did not differ between treatment conditions (*P*_*PWM*_ > 0.1). Avoidant PD patients in OPC reported greater use of community health services relative to other PDs (*P*_*TT*_ < 0.05). Medication, GP, and community services costs were not influenced by Axis-I comorbidity (*P*_*PWM*_ > 0.1).

### The sum of costs and clinical gains over the trial period

The sum of health service costs (trial treatment and all additional health services) for each month and for the full 36-month trial period did not differ by treatment condition, and costs declined over time in both conditions (Table 5, section C, D, and Figure 1). Clinical gains were superior in OPC after 36 months (Table 6, section A, B, and Figure 1). However, investigations of the two major PD subgroups revealed considerable differences within the sample.

Cost differences were insignificant for borderline PD patients (Table 5, section C and D). Clinical improvement was found in both treatment conditions (Table 6, section A and B). At the final assessment (period 3), GAF scores were within mild impairment levels [mean GAF_borderlineOPC_: 65 (SD 2), mean GAF_borderlineSDC_: 61 (SD 6), *P*_*TT*_ *>* 0.05]. In both treatment conditions, 70% reported employment or study by 36 months (Table 2). The increase in the proportion employed or studying from baseline to 36 months was 16%.

Health service costs were considerably higher for avoidant PD patients in OPC, but clinical outcomes were also better (Figure 1). This was demonstrated by higher monthly health service costs over the full trial period and especially during period 1 (Table 5, section C), along with GAF improvements in the last phase of the trial (Table 6, section A). Non-clinical GAF levels were reached only in OPC [Period 3: mean GAF_avoidantOPC_: 66 (SD 0.1); mean GAF_avoidantSDC_: 53 (SD 3), *P*_*TT*_ < 0.05], and occupational status was also better for OPC patients at 36 months (Table 2). There was a 54% increase (baseline to 36 months) in the proportion of avoidant PD patients who were employed or studying in OPC. The corresponding increase in SDC was 31%. SDC was cheaper, but less effective, and the extra cost for each additional GAF point gained per avoidant PD patient in OPC would be 1092 euros (incremental cost per incremental effect).

Health service costs and GAF were not influenced by comorbidity of other specific PDs or other Axis-I or -II disorders (*P*_*PWM*_ > 0.1).

### Health service costs and functioning the year before the trial

In the year before the trial, the average monthly costs of all health services did not differ by treatment condition (*P*_*LM*_ > 0.1). These baseline assessments revealed high costs of health services, poor global functioning, considerable comorbidity, symptom distress, and interpersonal problems (Figure 1, Tables 1 and 4). Compared with the rest of the sample, borderline PD was associated with particularly high costs of emergency services [mean cost difference: euros 955 (SE 441), *P*_*LM*_ = 0.03]. Avoidant PD was associated with poorer global functioning [mean GAF difference: -2(SE 1), *P*_*LM*_ = 0.03], but there was no indication of extreme health service use before the trial. Neither comorbid mood or anxiety disorders, other specific PDs nor increasing extent of Axis-I or -II comorbidity had significant influence on health service costs or functioning (both *P*_*LM*_ >0.1).

## Discussion

Our main findings were:

1) The step-down format required little adjuvant health services and was therefore not the more expensive, but its clinical effectiveness depended on type of PD pathology.

2) There was no difference in the cost-effectiveness of step-down and outpatient treatment for patients with borderline PD.

3) Outpatient treatment was clinically effective for avoidant PD patients, but also the most costly for this PD subsample, owing to considerable adjuvant health services.

### Step-down treatment is recommendable for treatment of borderline PD

Before starting treatment, borderline PD patients had the highest emergency costs in the sample. This observation reflects the well-described trend of extensive health service use previously associated with borderline PD [6]. In the course of the trial, the cost effectiveness of the two treatments could not be distinguished. Similarly, a cost-effectiveness study from the Netherlands found both day-hospital and outpatient formats recommendable in treatment of cluster B disorders [28]. Our study suggests a greater potential for specialist outpatient treatments for borderline PD patients than might have been expected, but additionally, it confirms that such treatments do not necessarily have lower overall costs when all services are included. The step-down condition had comparable costs and represented a well-defined treatment in its own right with little need for adjuvant health services. The latter are obvious advantages for both the patient and healthcare provider.

### The flexible format of outpatient treatment

Clinical effectiveness after 36 months was generally good in the outpatient condition. Costs, however, were highly variable. The composition of treatment and adjuvant health-service resources depended on the outpatient therapist’s evaluation of the patient, and presumably, also on personal style, interest, and availability. This flexibility had remarkable consequences for patients with avoidant PD.

Avoidant PD patients characteristically reported low functioning, but moderate use of health services the year before the trial. Their health service costs early in the trial therefore represent a considerable cost expansion reflecting a broad range of health services, both “high-” and “low-cost” services. Costs remained higher than the rest of the sample throughout the trial, although they declined together with clinical improvements in later phases. We have not found any other studies that report health-service use among avoidant PD patients. In the previously mentioned cost-effectiveness study of cluster C PDs [29], the most intensive treatment formats (day-hospital and inpatient treatment) were more cost-effective than outpatient treatment. In light of the adjuvant health services, the contrast to our outpatient condition may not be so great. However, our results are based on a small sample, and their generalisability is therefore uncertain. We nevertheless find the expansion of health service costs noteworthy, as the trend was still evident in analyses excluding patients with extreme emergency costs.

Were the “additional benefits” of the more effective outpatient treatment worth the “additional cost” for avoidant PD patients? From an economic perspective, one may say that if the high costs or extra health services “invested” in the outpatient condition contributed to superior long-term clinical gains, the extra costs per GAF point gained from outpatient treatment, for each patient with avoidant PD, would be 1092 euros [41]. The societal costs of one year of disability would, by far, exceed the extra costs in the outpatient condition. It is therefore likely that the outpatient treatment would have been the more cost-effective if we had also included societal costs. However, we cannot say for certain that the costs invested had any relation to the later improvements. The observed overall intensification of health care use in the outpatient condition may reflect a psychological destabilisation among vulnerable avoidant PD patients. Final outcomes would then depend upon how this destabilisation was managed. One could speculate that the flexibility of the format in itself was an important clinical factor. An integrated and eclectic treatment approach closely attuned to the individual has indeed been promoted in the treatment of severe PD [42]. The present study, however, has not included data on treatment processes.

We conclude that the long-term clinical effectiveness of outpatient treatment was impressive. However, because of the small sample size and the complexity and variation of adjuvant health services, we cannot identify specific components of the outpatient condition recommendable for the treatment of avoidant PD.

### The step-down programme

In the step-down condition, costs were remarkably homogenous, independent of the patient’s type of PD or other comorbid Axis-I or -II disorders. Our trial clearly demonstrates how the expectedly, high costs of step-down treatment were offset by the considerably lower costs of additional health services. Emergency services were equally available for patients in the two comparison treatments. In a study of health service use costs, Chiesa et al. similarly demonstrated that reduced emergency costs gave overall cost savings for expensive, clinically effective, hospital-based PD treatment programmes over less specified, non-specialist psychiatric treatment [14]. In the present study, the question of cost-effectiveness is more complicated for patients with avoidant PD. The less costly step-down alternative had also the least effect.

Emergency services are expensive and reflect severe clinical hazards. It could be assumed that step-down treatment prevented emergencies, and in itself, this would be an important benefit. However, we found that the use of cheaper outpatient emergency services in period 1 was actually more frequent in the step-down condition, which could suggest that avoidant PD patients in step-down treatment also experienced insufficient psychological containment in the first phase of treatment. The dismaying results of step-down treatment for avoidant PD patients are based on a small sample. However, in recent five-year follow-up studies of large PD cohorts (n = 352 and n = 790), poor long-term functioning and prevailing symptom distress were demonstrated for avoidant PD patients after similar step-down treatment [27,43]. Thus, the traditional step-down approaches may not be cost-effective for patients with avoidant PD.

Our results highlight a need for further investigation of the therapeutic process for individuals with avoidant personality structures. Several recent studies have focused on therapist style, interventions, and treatment principles in individual therapy for these patients [25,44-49]. We have not found process studies from intensive step-down treatments or outpatient group psychotherapy. More profound investigations, including case studies or qualitative interviews, might provide a better understanding of elements of the step-down treatment or group psychotherapy in need of modification, or of elements of the outpatient format that are beneficial. One hypothesis is that the type of step-down treatment investigated in this study is too supportive for avoidant PD patients. Embedded in a Scandinavian welfare state, it might be that patients are not challenged enough and thus establish their avoidant-role behaviour also within the treatment programme.

### Strengths and limitations

The UPP project aimed to recruit individuals from a naturally treatment seeking population of poorly functioning PD patients. We found that the baseline levels of functioning and distribution of diagnoses described in this study were similar to those described in other naturalistic, clinical studies of PD patients in day-hospital, step-down, and inpatient treatments [27,50], thus indicating that the present study population is clinically representative.

In clinical PD research, attrition is a frequent problem. This was also the case in our study. The lowest response rate was at the last assessment, with 25% of patients missing. Mixed-model statistics are a recommended statistical method for analyses of longitudinal data with long follow-up periods, uneven time intervals, and unequal numbers at each assessment [38]. Individual change trajectories are based on all available data. In our study, the longitudinal analyses thus incorporate all 107 patients. In the Methods section, we elaborated on our investigations concerning the possible bias of missing data. Although attrition will always represent some uncertainty, we conclude that it did not cause any systematic bias in the present study.

The validity of the statistical model (how well the change model describes the actual change pattern in the data) is an important limitation. Modelling procedures in the present study are described in the subsection about statistics. We found that correlations between the mixed models’ predicted values and the samples’ mean values were all significant (*P* < 0.01), an indication of satisfactory validity.

Registration of health-service use was based on patient interviews, and could be biased by the quality of patients’ recollection. The accuracy of reported frequencies of GP consultations, use of other community services, and medications may have suffered. However, important differences were associated with the use of emergency services. It can be argued that the more exceptional and dramatic nature of such situations likely increases the probability that they were reported accurately.

It should also be emphasised that the “costs” in the present study included only health services and not societal costs, such as loss of productivity. Societal costs are likely to be high among poorly functioning patients. For avoidant PD patients in the present trial, the decrease of unemployment and disability was considerably greater in the outpatient than the step-down condition. A broader cost analysis that included societal costs would most likely have strengthened the present results of our study by demonstrating greater cost savings in the outpatient condition.

The use of observer-rated GAF as a measure of clinical outcomes can be a limitation. Evaluations before randomisation were performed by day-hospital staff. Later evaluations were performed by research fellows and cross-checked by a second evaluator. Although GAF reliability was low at baseline, it was high during later periods, and very high in the last period, where we also found significant differences between the treatment conditions. Unreliable GAF ratings were therefore not likely to have biased these results. Moreover, the second clinical outcome measure, the self-report of occupational functioning, corresponded well with the GAF results.

## Conclusion

This study highlights the importance of cost-effectiveness studies in the treatment of PDs. We found no significant difference of costs for efficient treatment of borderline PD in two different treatment formats. Patients with avoidant PD improved more in the outpatient format, although it was more costly in the initial phase due to high supplementary medical and mental health service costs.

## Competing interests

The authors declare no financial or non-financial competing interests.

## Authors’ contributions

TW and SK were responsible for the conception, design, recruitment, and randomisation procedures for this study. EAA and FGR were responsible for the acquisition of data. VH was responsible for the estimation of unit costs and contributed to arrangement and interpretation of cost data. EHK was responsible for interpretation of the data, all statistical analyses, and was the main author of the manuscript. All authors have contributed to manuscript revisions and have read and approved the final manuscript.

## Authors’ information

The Research Group at the Department for Personality Psychiatry, Oslo University Hospital, Oslo, Norway.

## Pre-publication history

The pre-publication history for this paper can be accessed here:

http://www.biomedcentral.com/1471-244X/13/315/prepub

## Supplementary Material

Additional file 1 **Chart 1.** Patient flow; step down (SDC) versus outpatient treatment (OPC). Click here for file
