# Supplementary figures and images for: Health service costs and clinical gains of psychotherapy for personality disorders: a randomized controlled trial of day-hospital-based step-down treatment versus outpatient treatment at a specialist practice
Source: BMC Psychiatry. 2013 Nov 22;13:315. doi: 10.1186/1471-244X-13-315 (PMC4222503; doi:10.1186/1471-244X-13-315)

**Chart 1: Patient flow; step down (SDC) versus outpatient treatment (OPC)**

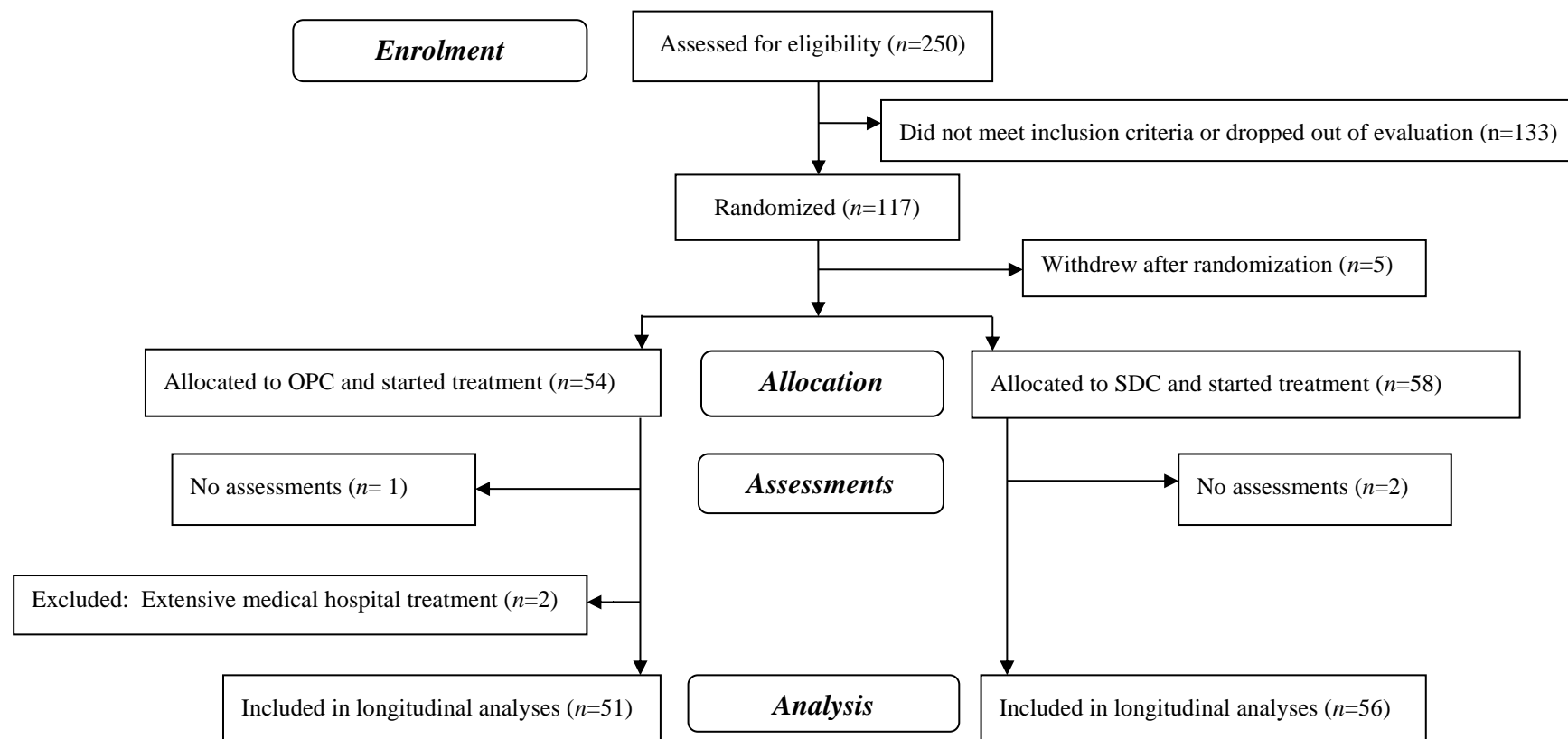

Supplement: Additional file 1 — Chart 1. Patient flow; step down (SDC) versus outpatient treatment (OPC). [file 1471-244X-13-315-S1.pdf]
